# Nationwide Cross‐Sectional Online Survey of Australian Clinicians' Pain Management Practices for Newborns During Heel Lance Procedures

**DOI:** 10.1002/pne2.70010

**Published:** 2025-07-04

**Authors:** Sophie Jones, Nicole Pope, Margaret Broom, Jeanie Cheong, Erin Church, Melinda Cruz, Christine East, Jillian Francis, Jade Ferullo, Andree Gamble, Priya Govindaswamy, Philippa Grimston, Rebecca Hyde, Kylie Pussell, Kaye Spence, Alicia Spittle, Linda Sweet, Amy Tagliante Saracino, Susan Walker, Jacquie Whitelaw, Denise Harrison

**Affiliations:** ^1^ School of Health Sciences University of Melbourne Parkville Victoria Australia; ^2^ Centre for Digital Transformation of Health University of Melbourne Parkville Victoria Australia; ^3^ Murdoch Children's Research Institute Melbourne Victoria Australia; ^4^ Royal Children's Hospital Melbourne Victoria Australia; ^5^ Child Health Evaluative Services, Research Institute The Hospital for Sick Children Toronto Ontario Canada; ^6^ Australian Capital Territory Government Canberra Australia; ^7^ The Royal Women's Hospital Melbourne Victoria Australia; ^8^ Mercy Hospital for Women Melbourne Australia; ^9^ Department of Obstetrics and Gynaecology University of Melbourne Parkville Victoria Australia; ^10^ NICU Lived Network Sydney Australia; ^11^ Judith Lumley Centre and School of Nursing and Midwifery La Trobe University Melbourne Australia; ^12^ Curtin School of Nursing Curtin University Perth Western Australia Australia; ^13^ Nursing and Midwifery Monash University Melbourne Australia; ^14^ Children's Hospital at Westmead Westmead New South Wales Australia; ^15^ The University of Sydney Sydney New South Wales Australia; ^16^ Miracle Babies Foundation Moorebank New South Wales Australia; ^17^ Western Sydney University Sydney Australia; ^18^ School of Nursing and Midwifery Deakin University Melbourne Victoria Australia; ^19^ Western Health Melbourne Victoria Australia; ^20^ University of Ottawa Ottawa Ontario Canada

## Abstract

The analgesic effects of breastfeeding (BF), skin‐to‐skin care (SSC), and oral sucrose/glucose for neonates during painful procedures are well‐established. Although parents report wanting to comfort their babies during painful procedures, use of these strategies is inconsistent. This study investigated clinicians' support/use of BF, SSC and sucrose during newborn heel lance in Australia and perceptions of a clinician‐targeted video demonstrating how to perform heel lance while newborns were BF/SSC. A cross‐sectional online survey was conducted. Snowball sampling and distribution via partner organizations were used. Descriptive statistics and content analysis were used for quantitative and qualitative data, respectively. Respondents included 729 nurses, midwives, and phlebotomists, caring for healthy newborns (39%, *n* = 283); sick newborns (41% *n* = 300) and both sick and healthy newborns (20%, *n* = 146). Most respondents caring for healthy newborns were “very likely” to support BF (80%, *n* = 199) and SSC (65%, *n* = 162). Most (89%, *n* = 237) caring for sick newborns were “very likely” to use sucrose; one third “very likely” to support mothers to BF (29%, *n* = 78) and 32% (*n* = 85) to use SSC. Barriers to BF and SSC included parents being absent and critically ill newborns. Most considered the video applicable (81%, *n* = 488) and likely to increase BF or SSC (84%, *n* = 502). Analysis from comment data identified two categories: “healthcare context and practice” and “parent and baby.” The key findings that clinicians reported the video to be highly useful and that BF and SSC during heel lance for healthy newborns was high confirm that further research is needed to examine parents' use of BF and SSC during painful procedures.

**Abbreviations**
ABA: Australian Breastfeeding Association
BF: Baby‐Friendly Health Initiative
HL: heel lance
NICU: Neonatal Intensive Care Unit
RM: registered midwife
RN: registered nurse
SCN: Special Care Unit
SSC: skin‐to‐skin care

## 1 Introduction

Almost all newborns have blood tests/needle procedures for routine newborn screening.

Sick infants in Neonatal Intensive Care Units (NICU) undergo multiple blood collections and other painful procedures during hospitalization [1]. Although essential, these procedures result in pain and distress for newborns, with physiological changes (elevated heart rate, respiratory rate, and reduced oxygen saturation) during the procedure, and parental anxiety and distress [1, 2]. The number of painful procedures is an important predictor of long‐term adverse outcomes, affecting brain development as well as developmental and cognitive outcomes in premature infants [3, 4]. Prevalence studies demonstrate that sick hospitalized neonates can undergo up to 17 painful procedures each day, often without effective pain management [1, 5, 6].

High‐quality synthesized evidence demonstrates the analgesic effects of breastfeeding (BF) [7], skin‐to‐skin care (SSC) (holding babies directly against a caregiver's unclad chest) [8], and small volumes of oral sucrose or glucose during painful procedures [9]. These strategies are safe, cost‐effective, simple to use, and widely recommended in clinical practice guidelines [10]. Parents report wanting to be present to comfort their babies during painful procedures [11, 12, 13, 14], preferably by BF or holding SSC [15]. Despite the evidence, recommendations, and parental preference, reports show these strategies are infrequently used in clinical practice [5, 6, 15, 16]. This highlights a critical knowledge‐to‐action gap that requires attention to address unnecessary pain and distress and ensure parents have rightful opportunities to be present and comfort their babies during painful procedures. Clinicians' support of parents to BF and hold SSC during non‐urgent painful procedures, and current practices regarding use of sucrose in Australian newborn care settings are, however, unknown, with the last data reported in 2011 [17].

A key barrier reported by nurses and midwives, to supporting BF or SSC during painful needle procedures is a lack of knowledge about best ergonomics—that is, how clinicians should position themselves during the needle procedures [18, 19, 20, 21, 22]. In response to this knowledge gap, parents and researchers co‐produced a four‐minute “ergonomics” video targeted at clinicians, titled BeSweet2Babies Ergonomic video (“Ergonomics video”) [23]. The video demonstrates evidence‐based ergonomic practices for clinicians while they perform newborn blood tests while babies are being BF or held SSC. The video was shared on social media sites in August 2019 and is publicly available on YouTube. However, this video has not been formally evaluated for implementation effectiveness (applicability, acceptability, feasibility, potential effectiveness) [24] nor has it been evaluated to determine if the content addresses the lack of clinician knowledge barrier. Evaluation within Australia is also required given that the video is being promoted within Australian healthcare contexts.

This study aimed to (1) examine current pain management practices during heel lance (HL) for newborns in Australia; (2) explore the perceived usefulness and applicability of the ergonomics video among nurses, midwives, and phlebotomists; (3) explore the facilitators and barriers to involving parents in neonatal pain management strategies; and (4) identify whether practices and perceptions of parent involvement in neonatal pain management differed for clinicians caring for healthy newborns compared with those caring for sick newborns.

## 2 Methods

A descriptive, cross‐sectional online survey hosted on the Qualtrics (Qualtrics, Provo, UT, USA) platform was used to collect quantitative and qualitative information from nurses, midwives, and phlebotomists. The study is reported in accordance with the Checklist for Reporting Results of Internet E‐Surveys (CHERRIES) [25]. Ethics approval for the study was obtained from the University of Melbourne Human Research Ethics Committee (ID number 2022‐23533‐32 124‐3).

### 2.1 Participants and Setting

The anonymous online survey was distributed via email by representatives from two professional nursing organizations, the Australian College of Neonatal Nurses, the Australian College of Midwives, the Australian and New Zealand Neonatal Network, a collaborative network, and via a peak body for breastfeeding education and support, the Australian Breastfeeding Association (ABA). The email included study information, electronic consent, and a survey link. Participants were eligible to complete the survey if they were English‐speaking, working clinically as a registered nurse, registered midwife, or phlebotomist in a NICU, special care nursery (SCN) or maternity unit in Australia. A secondary snowball strategy included emails from the research team to affiliated organizations and colleagues and postings on social media platforms (Twitter/X and LinkedIn). The survey was promoted during the ABA Health Professional Seminar Series held in Sydney, Melbourne, Brisbane, and Perth in March 2023. The survey was open between October 2022 and June 2023.

### 2.2 Survey Development

Canadian‐based researchers developed the survey, which has been piloted and used in previous studies [21, 22, 26]. To ensure contextual relevance and appropriateness, the survey was modified to include phlebotomists and subsequently re‐piloted in the present study by a panel of 14 stakeholders (nurses, midwives, phlebotomists, parents, and clinician researchers). This led to the rewording of several items to enhance clarity. These pilot data were not included in the results.

The final survey comprised 37 questions that included demographic information (academic qualifications, certifications, primary role and setting, years of experience, employment status, and affiliations with professional organizations). Initial questions asked about the respondents' knowledge and use of pain management strategies when performing non‐urgent HL on newborns. Respondents were then invited to watch the ergonomics video and answer questions about their previous viewing and perceptions of the video. Using 5‐point Likert scales, questions were asked about the likelihood of using each pain management strategy (very likely, likely, neutral, unlikely, very unlikely), frequency of parents requesting the strategies be used (always, often, sometimes, rarely, never), and applicability of the video (very applicable, applicable, neutral, not applicable, not applicable at all). Participants were asked to select barriers and facilitators to using BF and SSC from a list, which were based on those previously identified [22]. Participants were invited (in free‐text response boxes) to comment on the video or any aspects of neonatal pain management practices during HL. A copy of the survey is provided in the supplementary file.

### 2.3 Quantitative Data Management and Analysis

Quantitative data were exported into Microsoft Excel (Version 1908, 2022, Washington) and Statistical Package for Social Science (Version 27, 2020, IBM, New York) for analysis directly from Qualtrics XM. Qualitative data were managed and indexed in NVivo (Version 10, 2014, QRS International, Denver, Colorado).

Descriptive statistics (frequency and percentage) were performed to analyze survey responses, including participant demographic characteristics, current practices, perceptions of the videos, barriers, and facilitators to the use of BF and SSC. The use of BF, SSC, and sucrose, and the barriers and facilitators to their use for newborn pain were analyzed across the three participant groups (those caring for sick newborns, those caring for healthy newborns, and those caring for both sick and healthy newborns). Categorical variables relating to practices or perceptions reported by those caring for sick newborns, those caring for healthy newborns, and those caring for both healthy and sick newborns were compared using the chi‐squared test. *p* values < 0.05 were considered statistically significant.

### 2.4 Qualitative Data Analysis

Content analysis was undertaken on the qualitative data from the free‐text response questions [27]. In a series of steps, two authors (N.P. and S.J.) first familiarized themselves with the data by repeated reading of the survey responses. They developed a coding matrix to facilitate initial descriptive open coding. Data were analyzed collectively for the whole sample of participants working across the three settings (those caring for sick newborns, those caring for healthy newborns, and those caring for both sick and healthy newborns). N.P. and S.J. met regularly to discuss early analysis patterns, and codes were subsequently clustered together into candidate categories. Codes, candidate categories, and illustrative quotes were reviewed and refined in collaborative discussions between N.P., S.J., and D.H. Categories were then refined to determine the final categories and named with input from the full research team. Categories incorporating participant quotes are reported. Qualitative data were edited only for major spelling and grammatical errors.

## 3 Results

The survey link was accessed by 842 people across all Australian states and territories. Of these, 755 (89.7%) provided consent, with 729 (96.6% of those who consented) completing the survey. As not all respondents answered all the questions, exact numbers for presented data are stated.

### 3.1 Sample Characteristics

Three hundred (41%) respondents cared for sick newborns in a NICU/SCN, 283 (39%) cared for healthy newborns in a maternity unit, and 146 (20%) worked with healthy and sick newborns across both types of units. The roles of respondents, highest academic qualification, state of primary workplace, and current work status are presented in Table 1.

**TABLE 1:** Demographic characteristics.

	*N* (%)
Group of newborns primarily cared for	*N* = 729
Healthy newborns in a maternity unit	283 (38.8)
Sick newborns in Neonatal Intensive Care, High Dependency Unit or Special Care Nursery	300 (41.2)
Both healthy and sick newborns across maternity and neonatal units	146 (20)
State/territory of primary workplace	*N* = 591
Victoria	225 (38)
New South Wales	116 (19.6)
Queensland	134 (22.7)
Australian Capital Territory	18 (3.0)
South Australia	30 (5.1)
Western Australia	37 (6.3)
Tasmania	25 (4.2)
Northern Territory	6 (1)
Role description	*N* = 591
Registered nurse	199 (33.7)
Registered nurse/registered midwife	186 (31.5)
Registered midwife	153 (26)
Lactation Consultants/Registered nurse/registered midwife	25 (4.2)
Phlebotomist	9 (1.5)
Enrolled nurse	7 (1.2)
Student midwife	5 (0.8)
Doctor	5 (0.8)
Nurse practitioner	2 (0.3)
Highest academic qualification	*N* = 591
Certificate III	11 (1.9)
Bachelor of Nursing and/or Bachelor of Midwifery	227 (38.4)
Post Graduate Diploma/Certificate	238 (40.3)
Master by Coursework or Minor Thesis	79 (13.4)
Master of Philosophy (research thesis)	5 (0.8)
PhD/Clinical Doctorate	3 (0.5)
Other	28 (4.7)
Current employment status	*N* = 594
Casual	54 (9.1)
Part‐time	263 (44.3)
Full‐time	277 (46.6)

### 3.2 Current Practices

The use of BF, SSC, and sucrose reported by respondents across neonatal settings is presented in Table 2. Most of the 249 respondents caring for healthy newborns and those caring for both healthy and sick newborns (*n* = 135) reported they were very likely to support mothers to BF (80%, *n* = 199 and 60%, *n* = 81, respectively) and to hold their newborn SSC (65%, *n* = 162 and 59%, *n* = 80, respectively) during HL. Comparatively, 89% (*n* = 237) of 266 respondents caring for sick newborns reported they were very likely to use sucrose, but only one‐third indicated they were very likely to support mothers to BF or hold their newborn SSC (29%, *n* = 78, and 32%, *n* = 85, respectively). Reported support of fathers/partners to hold newborns SSC during HL was low across both settings; only 31% (*n* = 76) of healthy newborns and 21% (*n* = 56) of staff caring for sick newborns. Facilitating other family members or caregivers to hold SSC during HL was even less common; only 21% (*n* = 53) and 12% (*n* = 32) of respondents caring for healthy and sick newborns, respectively, reported they were very likely to do this. The setting in which participants practiced (caring for sick newborns, caring for healthy newborns or caring for both healthy and sick newborns) was associated with the likelihood of clinicians facilitating BF for a mother or SSC for father or partner/other caregiver (*X* ^2^, df 8, all *p* < 0.001) Clinicians caring for healthy newborns were more likely to facilitate BF (*X* ^2^, df 1, *n* = 484, *p* < 0.001) and SSC (*X* ^2^, df 1, *n* = 459, *p* < 0.001) for mothers and partners during HL compared to clinicians caring for sick newborns. Clinicians caring for sick newborns were more likely to administer sucrose for HL, compared to clinicians caring for healthy newborns (*X* ^2^, df 1, *n* = 478, *p* ≤ 0.0001).

**TABLE 2:** Current practices.

	Very likely	Likely	Neutral	Unlikely	Very unlikely	Total *N*
Facilitate mothers to breastfeed their newborn—*n* (%)						
Healthy newborns in a maternity unit	199 (79.9)	31 (12.4)	7 (2.8)	9 (3.6)	3 (1.2)	249
Sick newborns in a neonatal unit	78 (29.3)	73 (27.4)	24 (9.0)	63 (23.7)	28 (10.5)	266
Both healthy and sick newborns across maternity and neonatal units	81 (60)	33 (24.4)	8 (5.9)	11 (8.1)	2 (1.5)	135
Total	358 (55.1)	137 (21.1%)	39 (6.0)	83 (12.8)	33 (5.1)	650
Facilitate mothers to hold their newborn skin‐to‐skin—*n* (%)						
Healthy newborns in a maternity unit	162 (65.1)	47 (18.9)	20 (8.0)	16 (6.4)	4 (1.6)	249
Sick newborns in a neonatal unit	85 (32.0)	72 (27.1)	36 (13.5)	53 (19.9)	20 (7.5)	266
Both healthy and sick newborns across maternity and neonatal units	80 (59.3)	31 (23.0)	7 (5.2)	14 (10.4)	3 (2.2)	135
Total	327 (50.3)	150 (23.1)	63 (9.7)	83 (12.8)	27 (4.2)	650
Facilitate fathers to hold their newborn skin‐to‐skin—*n* (%)						
Healthy newborns in a maternity unit	76 (30.5)	65 (26.1)	57 (22.9)	33 (13.3)	18 (7.2)	249
Sick newborns in a neonatal unit	56 (21.1)	69 (25.9)	48 (18)	66 (24.8)	27 (10.2)	266
Both healthy and sick newborns across maternity and neonatal units	49 (36.3)	34 (25.2)	22 (16.3)	21 (15.6)	9 (6.7)	135
Total	181 (27.8)	168 (25.8)	127 (19.5)	120 (18.5)	54 (8.3)	650
Facilitate others (partners, family members, other caregivers) to hold their newborn skin‐to‐skin—*n* (%)						
Healthy newborns in a maternity unit	53 (21.3)	46 (18.5)	49 (19.7)	72 (28.9)	29 (11.6)	249
Sick newborns in a neonatal unit	32 (12)	36 (13.5)	53 (19.9)	73 (27.4)	72 (27.1)	266
Both healthy and sick newborns across maternity and neonatal units	35 (25.9)	27 (20)	26 (19.3)	25 (18.5)	22 (16.3)	135
Total	120 (18.5)	109 (16.8)	128 (19.7)	170 (26.2)	123 (18.9)	650
Administer sucrose—*n* (%)						
Healthy newborns in a maternity unit	43 (17.3)	46 (18.5)	33 (13.3)	67 (26.9)	60 (24.1)	249
Sick newborns in a neonatal unit	237 (89.1)	23 (8.6)	4 (1.5)	1 (0.4)	1 (0.4)	266
Both healthy and sick newborns across maternity and neonatal units	63 (46.7)	27 (20)	13 (9.6)	20 (14.8)	12 (8.9)	135
Total	343 (52.8)	96 (14.8)	50 (7.7)	88 (13.5)	73 (11.2)	650

**FIGURE 1:**
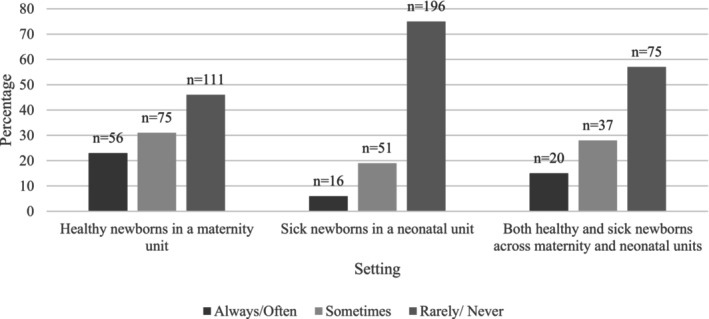
Clinicians' perceptions of mothers' requests to BF during HL.

**FIGURE 2:**
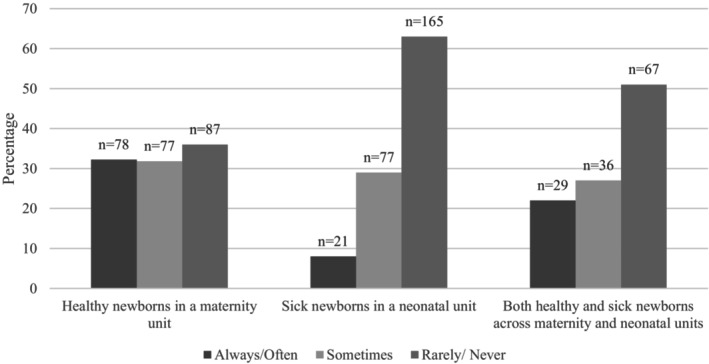
Clinicians' perceptions of mothers' requests for SSC during HL.

**FIGURE 3:**
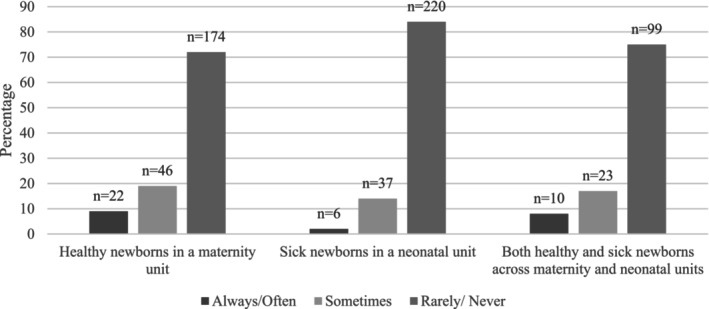
Clinicians perceptions of partners' requests for SSC during HL.

Results for clinicians' perceptions of parents advocating for pain management by requesting to either BF or hold SSC or partners advocating for mothers to BF during HL, differed across settings. As illustrated in Figures 1, 2, 3, of the 242 clinicians caring for healthy newborns who answered this question, 46% (*n* = 111) reported mothers “rarely/never” request BF, 36% (*n* = 86) reported mothers “rarely/never” request SSC, 78% (*n* = 188) reported partners “rarely/never” request the mother to BF, and 72% (*n* = 174) reported partners “rarely/never” request SSC. Respondents caring for sick newborns (*n* = 263) reported most mothers “rarely/never” (75%, *n* = 196) request to BF and “rarely/never” (63%, *n* = 165) request to hold their baby SSC. These differences in respondents' perceptions of requests for BF or SSC by mothers between the two groups (those caring for healthy newborns and those caring for sick newborns) were statistically significant (*X* ^2^, df 2, *n* = 505, *p* < 0.001). The majority of respondents caring for sick newborns indicated that fathers/partners “rarely/never” (92%, *n* = 242) request for the mother to BF or “rarely/never” (84%, *n* = 220) request to hold their newborn SSC. The difference between respondents' perceptions of fathers'/partners' request for the mother to BF or to hold their newborn SSC, comparing those caring for healthy newborns and those caring for sick newborns, was also significant (*X* ^2^, df 2, *n* = 505, *p* < 0.001).

### 3.3 Facilitators to the Use of Breastfeeding and Skin‐to‐Skin Care During Heel Lance

**FIGURE 4:**
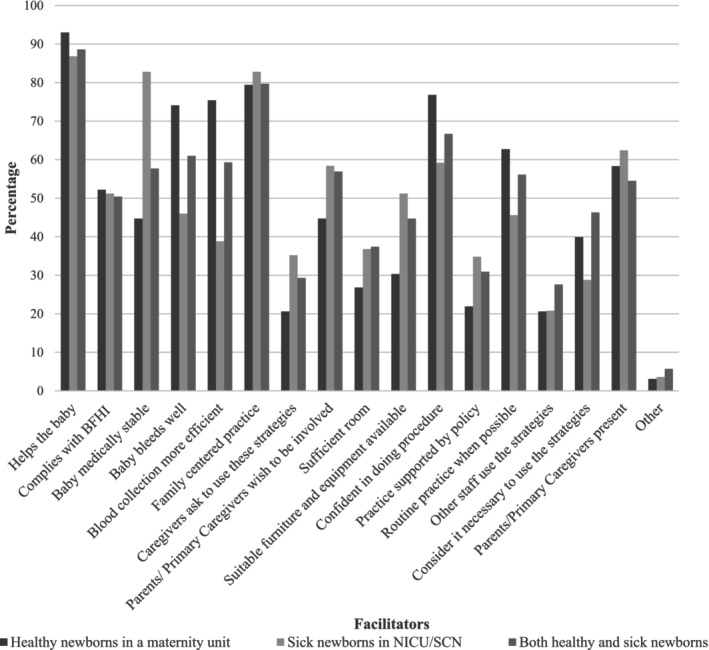
Facilitators to using BF and SSC for neonatal pain management.

A perception that BF and SSC “helps the baby” during HL was commonly identified as a facilitator to using these strategies by the majority of respondents caring for newborns in all settings. Figure 4 presents responses from the facilitators to BF and SSC during HL for respondents caring for healthy newborns, respondents caring for sick newborns and respondents caring for both healthy and sick newborns. Other facilitators identified by more than 50% of respondents from each setting include that BF and SSC comply with the Baby‐Friendly Health Initiative, family‐centered practice, clinician confidence with the procedure, and the presence of the parent/primary caregiver.

### 3.4 Barriers to the Use of Breastfeeding and Skin‐to‐Skin Care During Non‐Urgent Heel Lance

**FIGURE 5:**
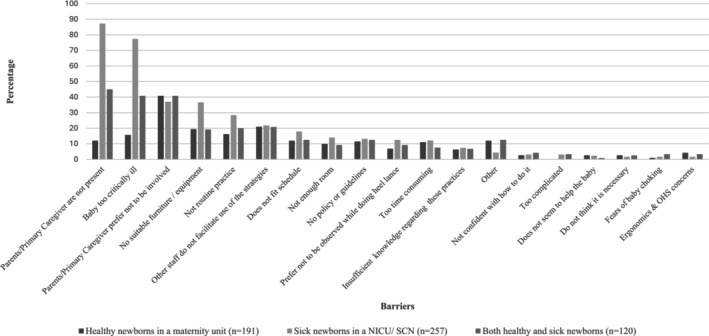
Barriers to using BF and SSC for neonatal pain management.

A total of 568 respondents selected barriers hindering clinicians from facilitating BF or SSC during HL (Figure 5). The top five barriers to BF or SSC during HL selected by clinicians caring for healthy newborns were (1) parents preferred not to be involved (41%, *n* = 78); (2) other staff do not facilitate the use of the strategies (21%, *n* = 40); (3) no suitable furniture/equipment available (19.4%, *n* = 37); (4) not routine practice to do so (16.2%, *n* = 31); and (5) baby too critically ill (15.7%, *n* = 30).

The top five barriers selected by clinicians caring for sick newborns and by those caring for both healthy and sick newborns were the same. These included (1) parents/primary caregivers not being present during HL (87%, *n* = 224 and 45%, *n* = 54, respectively); (2) baby too critically ill (77.4%, *n* = 199 and 41%, *n* = 49, respectively); (3) parents prefer not to be involved (37%, *n* = 95 and 41%, *n* = 49, respectively); (4) no suitable furniture/equipment (36.6%, *n* = 94 and 19%, *n* = 23, respectively); and (5) and not routine practice (28.4%, *n* = 73 and 20%, *n* = 24, respectively).

### 3.5 Perceptions of the Ergonomics Video

Only 101 (15.6%) of 647 respondents had seen the ergonomics video prior to participating in the study. Of these, most had previously seen the video at a conference (30%, *n* = 30) or on YouTube (22%, *n* = 22). Most respondents reported that the video was “very applicable” or “applicable” to their practice (80.9%, *n* = 488) and that the length of the video was “very acceptable” or “acceptable” (89.7%, *n* = 541). All clinicians caring for healthy newborns reported being more likely to facilitate BF after viewing the ergonomics video (*n* = 223, 100%), as did most clinicians caring for sick newborns (*n* = 207, 91%). Most respondents (84%, *n* = 502) reported that the use of the video would “very likely” or “likely” increase BF or SSC during HL procedures, with no differences between the different settings (*p* = 0.2). Similarly, most respondents (83%, *n* = 497) from all settings reported they would “very likely” or “likely” recommend the use of the video to other staff.

### 3.6 Qualitative Results

A total of 322 participants made comments that were included in the qualitative data analysis. Two broad categories were developed from the content analysis of the text data: (1) the healthcare context and clinical practice and (2) parent and baby.

#### 3.6.1 Category 1: The Healthcare Context and Clinical Practice

This category related to the environmental context and resources that influenced the use of pain management strategies. Having well‐staffed clinical units facilitated the use of the strategies. Material resources such as adjustable beds and tables to support safe, ergonomic practices were acknowledged as important facilitators to using the strategies, but these were not always available, especially in community care.

It's a great idea, but unfortunately, many of the units I have worked in require me to squat on the floor at a terrible angle in order to facilitate it. (Participant 196, caring for sick newborns)

Having adequate space to perform the procedure was also an important factor in using pain management strategies. However, respondents reported that often, there was not enough space to maneuver and perform the procedures safely and effectively and to make the parent/caregiver feel comfortable being involved during painful procedures.

It is difficult to get Mum to the correct height without a bed. I do try to sit on a height‐adjustable stool, but it is still hard to get close enough to sit up straight. (Participant 237, caring for sick newborns)

We have very limited space in our SCN and NICU. (Participant 162, caring for sick newborns)

Respondents highlighted that clinical practice guidelines included recommendations for using pain management strategies during HL and that the strategies were already routinely used in daily practice.

I have used all these procedures for 25yrs as per hospital policy!!! (Participant 270, caring for healthy newborns)

Most nurses and midwives I work with know the benefits of babies having skin‐to‐skin or breastfeeding during heel prick blood tests. And I feel that most put this into practice when suitable. (Participant 146, caring for sick newborns)

Respondents reported that the video was most relevant in postnatal clinical contexts where babies are medically stable and parents are present. In these cases, the video was considered a useful teaching tool, especially for students or junior midwives.

This video would be ideal to be shown to midwifery students so they can facilitate this practice from day dot. (Participant 152, caring for sick newborns)

However, many survey comments demonstrate that clinicians had not known about or seen the video prior to this study.

Have never heard of this video prior to the survey, you need to explore how to promote its existence. (Participant 118, caring for healthy newborns)

Respondents suggested integrating the video into staff orientation programs or in‐service education to increase its visibility. Others noted that the video could be used as an educational tool for parents.

[The video] should be part of antenatal education. (Participant 293, caring for healthy and sick newborns)

Many of the comments demonstrated that respondents had positive sentiments about the video; “Great video. Simple and practical” “Clear resource” “Easy watching”. Even so, respondents suggested that the video could be shorter in duration and modified with content that was more relevant to other clinical contexts and cultures.

The video has no relevance to midwives doing blood collection at home. For early discharge from hospital women, this is done in the home by hospital‐based midwives and continuity of midwifery practice midwives. For these reasons, I would not recommend the video to my colleagues. (Participant 131, caring for healthy newborns)

It would be good to have examples of how to facilitate this with a father holding [the baby] in a chair. (Participant 230, caring for healthy newborns)

Many respondents noted their routine use of pain management strategies in their practice. The video was regarded as a useful tool to address skills and knowledge gaps among staff who lacked experience and specialized skills, such as students or novice clinicians.

Would benefit new graduates to gain confidence. For some it would encourage this as expected practice. (Participant 39, caring for healthy newborns)

#### 3.6.2 Category 2: Parent and Baby

The babies' clinical state was one of the most noted barriers to using the strategies. Babies were often considered too unwell or unstable for these strategies to be used as safe and feasible options during HL.

I work in a NICU and we always use sucrose, however our babies are too small or sick to breastfeed and only come out for cuddles when stable, we would not take baby out of their cot for a blood test as it would be too disruptive. (Participant 170, caring for sick newborns)

I work with extremely preterm babies that are usually critically unwell. BF is not possible and skin to skin for pain related procedures is often not possible. (Participant 189, caring for sick newborns)

Parental/caregiver presence was another important factor that influenced the use of the strategies. When parents were not at the bedside, whether due to parental preference, their caring and work responsibilities, or the ward routine, the strategies were not used.

I most often get women to breastfeed their baby while doing the bloods, but sometimes parents don't want to be involved because they fear watching the baby in pain. (Participant 264, caring for healthy newborns)

Whenever parents are present, I facilitate these practices; in the situation when parents are not present, i.e., during night shift hours, I then use sucrose. (Participant 152, caring for sick newborns)

Parents do not always feel comfortable in using these strategies. (Participant 13, caring for healthy newborns)

Blood collection does not coincide with parents being present. In the neonatal unit, bloods are time relevant and cannot be held until parents visit. (Participant 68, caring for healthy newborns)

## 4 Discussion

Findings of this Australian nationwide multi‐method clinician‐targeted online survey of pain management practices during newborn HL and evaluation of the ergonomics video highlight differences between pain management practices for sick and healthy newborns. The high reported use and support of BF and SSC during HL by clinicians caring for healthy newborns demonstrate widespread use of the evidence for healthy newborns. For sick newborns, the reported use of sucrose in NICUs during painful procedures is higher than that reported in recently published prevalence studies [16, 26, 28, 29], which may indicate either high adoption of sucrose for analgesia in Australian neonatal settings or differences in perceived practices versus actual practices. The frequent use of sucrose for newborns in NICU in this study may also be influenced by nurses and midwives with an interest in pain management self‐selecting to participate in the study. Thus, those with an interest in pain management may be more likely to report use of pain management strategies.

Involving the families in their newborn's pain management during HL relies on both clinicians and families being informed, aware, and able to advocate. Therefore, if families are unaware that BF and SSC are effective pain management strategies, they cannot request to BF or hold their newborns SSC during painful procedures. Additionally, the clinicians' perception that parents prefer not to be involved is in contrast to that reported by parents themselves, who do wish to be involved [12, 15].

There are numerous studies included in systematic reviews describing the development and evaluation of e‐health resources targeted at parents of infants to inform them of procedural pain management [30, 31, 32]. Richardson et al. [32] included 11 studies evaluating parent‐targeted videos published since 2013. Such resources aim to inform and help parents to advocate for themselves and their newborns. However, such resources are not always found and viewed by parents. In a survey conducted in Australia, including parents of sick newborns, most respondents had not previously seen a publicly available video specifically targeted at parents [33] showing BF and SSC occurring during HL [15]. In addition, most parents did not know that these strategies could be used, and most had not previously been involved during painful procedures [15]. In another study, using the same parent‐targeted educational video [33], parents of healthy newborns in one site in Canada were shown the video prior to their newborn's HL for newborn screening [34]. Despite most parents reporting their preferences to use BF and SSC during HL, these strategies were rarely used. Instead, clinicians more frequently used sucrose, or no pain management strategy was utilized. In a study that included nurses' and parents' perceptions of pain management in the NICU, nurses perceived they supported parents during painful procedures more frequently than parents perceived being supported by nurses [35]. Most parents of preterm newborns in a study conducted in France reported not being informed about their possible role in pain management [36] and other reports demonstrate limited involvement of parents in neonatal pain management [5, 6, 13, 18, 31, 34]. Such studies highlight that both parents of newborns and clinicians need to be informed, empowered, and facilitated to use these strategies [30], especially as parents report wanting to be involved [11, 12, 13, 14, 15]. Yet, this conflicts with results from the current study, where respondents' perception was that parents do not want to and should not be involved as it could cause the parent further distress.

The ergonomics video evaluated in this study was produced as a result of clinicians reporting barriers to using BF or SSC during HL, related to ergonomics, that is, clinicians knowing how to position themselves and the parent during the procedure [18, 19, 22, 37]. Despite most of the respondents not viewing the video prior to this study, the responses to the video questions showed that the video was perceived to be highly applicable, “very likely” or “likely” to be recommended to other staff and likely to increase the use of BF and SSC for future painful procedures. These results are promising; however, the fact that most nurses had not previously seen the video before this study highlights that the video is not reaching target end‐users.

### 4.1 Barriers and Facilitators

Respondents who primarily cared for sick newborns reported barriers to using BF or SSC more frequently. The top barrier selected was that the parent(s) were not present. This was also reported by Benoit et al. [18]. Despite efforts to promote family‐centred care and support parents being present and involved in all aspects of NICU care [38], facilitating parental presence during routine blood collection remains challenging [18]. Many of the respondents' comments also related to parents not being present in the unit when blood tests were performed. Although the wording in the survey specified pain management during non‐urgent HLs, which could potentially be planned around parents' ability to be present, this planning does not occur consistently. This may relate to the parents' ability to be present [18, 37] as well as to the NICU culture and clinicians' preferences to perform procedures without parents [21, 39]. Other barriers to involving parents in pain management, including workload issues, have also been reported [18, 22, 40].

Our sample of respondents included clinicians caring for both sick and healthy newborns and included a small number of phlebotomists, which limited direct comparison of responses. There is little published information about phlebotomists' newborn pain management; however, one report of a quality improvement initiative to improve ergonomics for staff performing HL during newborn screening included phlebotomists along with nurses [37], while another study demonstrated that phlebotomists do not see pain management as their role and prefer not to involve parents [3]. However, as phlebotomists play key roles in many neonatal settings, their inclusion in any pain management education decisions and initiatives is vital.

### 4.2 Strengths and Limitations

As snowball sampling was used via social media and other platforms, the response rate for the survey cannot be calculated. However, a strength of this study is the number of responses from diverse locations around Australia. These data, therefore, inform current newborn pain management practices during HL, by clinicians in Australia. However, low numbers of responses from some states and territories, especially the Northern Territory, with only 1% of total responses, limit the generalisability of the findings. Additionally, the timing of the survey coincided with the end of the COVID‐19 pandemic, where research priorities in healthcare settings were often directed toward the impacts of COVID‐19. This period also exposed vulnerabilities of a burdened workforce, which may account for the limited uptake of the survey and potentially impact clinicians' opportunities to watch the video, especially in the less populated states and territories. Finally, relying on self‐report data is a limitation.

## 5 Conclusions

Clinicians' reported use of BF and SSC during non‐urgent HL for healthy newborns was very high, even though clinicians reported that parents infrequently asked to use these strategies. The reported use of BF and SSC in sick newborns was much lower. Frequently reported barriers to involving parents in pain management for sick newborns were that parents were not present, and the baby was too critically ill. In contrast, the highest reported barrier for healthy newborns was that parents preferred not to be involved. The clinician‐targeted ergonomics video evaluated in this study was perceived to be highly applicable and useful in increasing the future use of BF and SSC during HL. Further evaluation of the video using behavioral outcome data is warranted to ascertain its effectiveness in improving pain management practices during HL in sick and healthy newborns. Future research is required to examine Australian parents and caregivers' current knowledge, experiences and use of BF, SSC, and sucrose for their newborn or infant during painful procedures.

## Author Contributions

Denise Harrison was responsible for study design, development of the survey, assisted in survey promotion, supervised data analysis and co‐wrote the manuscript. Sophie Jones contributed to the development of the survey, assisted in survey promotion, conducted the qualitative and quantitative data analyses, and co‐wrote the manuscript. Nicole Pope assisted with study design, contributed to the development of the survey, assisted in survey promotion, conducted the qualitative and quantitative data analysis and co‐wrote the manuscript. Margaret Broom, Jeanie Cheong, Erin Church, Melinda Cruz, Christine East, Jillian Francis, Jade Ferullo, Andree Gamble, Priya Govindaswamy, Philippa Grimston, Rebecca Hyde, Kylie Pussell, Kaye Spence, Alicia Spittle, Linda Sweet, Amy Tagliante Saracino, Susan Walker, Jacquie Whitelaw assisted in survey development, survey promotion and dissemination, and reviewed the manuscript. All authors approved the final manuscript as submitted and agree to be accountable for all aspects of the work.

## Conflicts of Interest

The authors declare no conflicts of interest.

## Supporting information


Data S1.

## Data Availability

The data that support the findings of this study are available on request from the corresponding author. The data are not publicly available due to privacy or ethical restrictions.
