# Supplementary material for: Nationwide Cross‐Sectional Online Survey of Australian Clinicians' Pain Management Practices for Newborns During Heel Lance Procedures
Source: Paediatr Neonatal Pain. 2025 Jul 4;7(3):e70010. doi: 10.1002/pne2.70010 (PMC12227018; doi:10.1002/pne2.70010)
Supplement: Supplementary file 1 — Data S1. [file PNE2-7-e70010-s001.pdf]

Partnering to reduce neonatal pain (ACM & ACNN Survey 2022)

Start of Block: Introduction & Instructions

Plain Language Statement

Project: Partnering to Reduce Neonatal Pain

Responsible Researcher:
Professor Denise Harrison
Department of Nursing, School of Health Sciences, Faculty of Medicine, Dentistry and Health Sciences, The University of Melbourne
Tel: 03 9035 8034
Email: deniseh@unimelb.edu.au

Additional Researchers:

Research team members include a consumer representative as well as researchers and clinicians from nursing, midwifery, medicine and allied health professions. Members are from various organisations including; The University of Melbourne; The Murdoch Children's Research Institute, Deakin University; The Royal Children's Hospital, Melbourne; The Royal Women's Hospital, Melbourne; Children's Hospital at Westmead, Sydney and Miracle Babies Foundation

Introduction

Thank you for your interest in participating in this research project. You have been invited to participate in this study because you are a member or on the mailing list of the Australian College of Neonatal Nurses (ACNN), the Australian College of Midwives (ACM) or you have identified yourself as eligible through our social media posts. The following page will provide further information about the project, so that you can decide if you would like to take part in this research.

Your participation is voluntary. If you do not wish to take part, you do not have to. If you begin participating, you can also stop at any time.

Please take the time to read this information. Please feel free to contact us with any questions.

What is this research about?

Healthy newborns require newborn screening and preterm and sick babies require repeated needle-related painful procedures.These procedures cause distress for at the time of procedure, and for sick preterm babies, repeated procedures increase risks of long-term developmental disabilities.

There are effective ways to reduce pain during heel lancing and other needle-related painful procedures. Breastfeeding, skin-to-skin against a parent’s chest, or giving small amounts of sugar water to suck, all effectively reduce pain. The strategies used depend on the health status of the baby and presence of the mother/other parent or close family/friend. These strategies are safe, simple to use, and recommended in international guidelines. Furthermore, parents report wanting to be involved and to comfort their baby during painful procedures. However, research shows these approaches are inconsistently used during painful procedures.

A key barrier reported by nurses and midwives to supporting breastfeeding or skin-to-skin during needles is a lack of knowledge about best ergonomics – i.e. how to position themselves during the needles. In response to this knowledge gap, Harrison and her team of parents, nurses, midwives, and an occupational health therapist co-produced an ‘ergonomics’ video – showing positioning for the nurse/midwife during the babies’ blood tests, while babies are breastfeeding or held skin-to-skin. This video was posted onto YouTube in 2019 for public viewing. However, we do not yet know if the video is being accessed by nurses and midwives in Australia, and how useful the video is for clinicians. Additionally, we do not have current data on frequency of use of these recommended pain management strategies.

Completion of this questionnaire will help determine current pain management practices and evaluate the potential usefulness of this educational video for Australian neonatal and maternity care nurses and midwives.

What will I be asked to do?

If you choose to participate in this research project, the following will happen:

You will be invited to watch a 4-minute online video.

You will then be invited to complete an online survey that asks about your perception of the video and about your perceptions and experiences with pain management strategies when performing heel lance procedures on newborn infants.

The total time commitment for participating in this research is about 10-15 minutes.

What are the possible benefits?

You will not benefit directly from participating. However, the information you provide may lead to future improvements in the way that nurses and midwives support families to help reduce their babies' pain during painful procedures.

What are the possible risks?

We do not foresee any risks associated with participating in this research project.

Do I have to take part?

Participation is totally voluntary. If you do not wish to take part, you do not have to.

If you decide to take part and submit your responses, but later change your mind, information that you have already provided in the survey will be included in our analysis. However responses you provide will not be identifiable.

Your decision about whether to take part or not to take part will remain anonymous to the researchers and to your employer.

How will I hear about the results of this project?

We will share the findings of this research via a summary of the survey results that will be disseminated to members of the ACNN and ACM.

We will also publish the results of the research through journal articles, conference presentations, and professional associations.

What will happen to information about me?

You are not required to provide any personal details in the survey. Your participation and the responses you provide are anonymous.

We intend to protect the confidentiality of your information within the limits of the law.

The information you provide will be treated as confidential and will be stored electronically and held under password protection at The University of Melbourne. The information you provide will be accessible only to Harrison (the responsible researcher) and her research assistants who will be working on data analysis. In accordance with research code requirements, we will store the information collected for 5 years after the release of any publications of this research project.

We may reuse the data we collect in this study for future research in the same general area of research as this project.

The data we collect in this project, and any future reuse of this data, will always be published as summary statistics, so your individual responses will never be identifiable or reported separately. If you decide to include potentially identifying comments in the survey, then if these comments are published, we will not include any potentially personally identifiable details.

Where can I get further information?

If you would like more information about the project, please contact the Responsible Researcher:

Professor Denise Harrison
Department of Nursing, Faculty of Medicine, Dentistry and Health Sciences,
The University of Melbourne
Tel: 03 9035 8034
Email: deniseh@unimelb.edu.au

Who can I contact if I have any concerns about the project?

This project has human research ethics approval from The University of Melbourne **[insert Project ID here]**. If you have any concerns or complaints about the conduct of this research project, which you do not wish to discuss with the research team, you should contact the Research Integrity Administrator, Office of Research Ethics and Integrity, University of Melbourne, VIC 3010. Tel: +61 8344 1814 or Email: research-integrity@unimelb.edu.au. All complaints will be treated confidentially. In any correspondence please provide the name of the research team and/or the name or ethics ID number of the research project.

Q3 If you wish to keep a copy of this Plain Language Statement, you can download it as a PDF below

End of Block: Introduction & Instructions

Start of Block: Consent

Consent form **Consent Form**

Department of Nursing; School of Health Sciences; Faculty of Medicine, Dentistry and Health Sciences

Project: Partnering to Reduce Neonatal Pain

Responsible Researcher:

Professor Denise Harrison
Department of Nursing, School of Health Sciences, Faculty of Medicine, Dentistry and Health Sciences, The University of Melbourne
Tel: 03 9035 8034
Email: deniseh@unimelb.edu.au

Co-principal researchers:

Rosemarie Boland
Linda Sweet
Leah Hickey
Melinda Cruz
Abbey Eeles
Amy Tagliante Saracino
Kay Spence
Jeanie Cheong
Alicia Spittle

Associate researchers:

Nicole Pope
Helena Bujalka

Consent

I consent to participate in this project, the details of which have been explained to me, and I have been provided with a written plain language statement to keep.

I understand that the purpose of this research is to determine the current pain management practices of nurses and midwives who care for sick and healthy newborns, and to evaluate the potential usefulness of a training video that demonstrates the positioning for the nurse/midwife during the babies' blood tests, while babies are breastfeeding or held skin-to-skin.

I understand that my participation in this project is for research purposes only.

I acknowledge that the possible effects of participating in this research project have been explained to my satisfaction.

In this project I will be required to complete an online survey about my perception of the video and about my perceptions of and experiences with pain management strategies when performing heel lance procedures on newborn infants.

I understand that my participation is voluntary and that I am free to withdraw from this project anytime without explanation or prejudice and to withdraw any unprocessed data that I have provided.

I understand that the data from this research will be stored at the University of Melbourne and will be destroyed 5 years after publication.

I have been informed that the confidentiality of the information I provide will be safeguarded

subject to any legal requirements; my data will be password protected and accessible only by the named researchers.

I understand that by checking the box below and responding to the survey, I consent to participate in this research.

Consent agreement Do you consent to participate in this survey?

- ◯ Yes, I consent to participate (1)
- ◯ No, I do not consent (2)

Skip To: End of Survey If Do you consent to participate in this survey? = No, I do not consent

---

Page Break

Q1 Which professional groups are you a member of?
Select all that apply.

- ☐ Australian College of Neonatal Nurses (ACNN) (1)
- ☐ Australian College of Midwives (ACM) (3)
- ☐ Perinatal Society of Australia and New Zealand (PSANZ) (7)
- ☐ Australian College of Children and Young Peoples Nurses (ACCYPN) (6)
- ☐ International Lactation Consultant Association (ILCA) (8)
- ☐ Lactation Consultants of Australia and New Zealand (LCANZ) (9)
- ☐ The Australian College of Critical Care Nurses Ltd (ACCCN) (10)
- ☐ I am not a member of any professional groups (5)
- ☐ Other: (please specify) (4) ____

Display This Question:

If Which professional groups are you a member of? Select all that apply. = Australian College of Neonatal Nurses (ACNN)

Q70 Are you a member of any ACNN special interest group (SIG)? If so, please select all that apply.

- ▢ Research SIG (1)
- ▢ Neurodevelopmental Care SIG (2)
- ▢ Education SIG (4)
- ▢ Infant Feeding SIG (5)
- ▢ Leadership SIG (6)
- ▢ Low Resource Countries SIG (7)
- ▢ Neonatal Skin SIG (8)
- ▢ Neonatal Nurse Practitioner SIG (9)
- ▢ I am not a member of any ACNN SIG (12)

End of Block: Consent

Start of Block: Branch logic for subgroups

Q2 Which group of newborns do you **primarily** care for?

- ○ Healthy newborns in a maternity unit (1)
- ○ Sick newborns in a neonatal unit (2)

End of Block: Branch logic for subgroups

Start of Block: video

Q3 Please answer the following based on your current knowledge;

Q4 a) Breastfeeding during needle related procedures reduces pain in newborns;

○ True (1)

○ False (2)

Q5 b) Skin-to-skin during needle-related procedures reduces pain in newborns;

○ True (1)

○ False (2)

Q6 c) Small volumes of sucrose or glucose during needle-related procedures reduces pain in newborns;

○ True (1)

○ False (2)

Q7 Please watch the Be Sweet to Babies 'Ergonomics' video below (which runs for 3:42 minutes) showing positioning for the nurse/midwife during the babies’ blood tests, while babies are breastfeeding or held skin-to-skin

---

Q8

Page Break

Q9 Had you seen the Be sweet to Babies 'Ergonomics' video before today?

- ◯ Yes (1)
- ◯ No (2)

Display This Question:

If Had you seen the Be sweet to Babies 'Ergonomics' video before today? = Yes

Q6 Where have you seen this video before? (Select all that apply)

- ▢ Facebook (1)
- ▢ Twitter (2)
- ▢ YouTube (3)
- ▢ Conference (4)
- ▢ University course (5)
- ▢ Webinar (6)
- ▢ Workplace (7)
- ▢ Not sure/can't remember (8)
- ▢ Other (please specify) (9) ____

Page Break

Page Break

Q10 Before viewing the video, how likely were you to;

	Very Likely (1)	Likely (2)	Neutral (3)	Unlikely (4)	Very unlikely (5)
a) Facilitate mothers to breastfeed their newborn during non-urgent heel lancing? (1)	o	o	o	o	o
b) Facilitate mothers to hold their newborn skin-to-skin during non-urgent heel lancing? (2)	o	o	o	o	o
c) Facilitate fathers to hold their newborn skin-to-skin during non-urgent heel lancing? (3)	o	o	o	o	o
d) Administer sucrose during non-urgent heel lancing? (4)	o	o	o	o	o

Page Break

Q11 How often do mothers request that they;

	Never (1)	Rarely (2)	Sometimes (3)	Often (4)	Always (5)
Breastfeed their baby during heel lance? (1)	○	○	○	○	○
Hold their baby skin-to-skin during heel lance? (2)	○	○	○	○	○

Q12 How often do partners request;

	Never (1)	Rarely (2)	Sometimes (3)	Often (4)	Always (5)
That mothers breastfeed their baby during heel lance? (1)	○	○	○	○	○
That they hold their baby skin-to-skin during heel lance? (2)	○	○	○	○	○

Page Break

Q13 This video was produced to address nurses’ and midwives’ reported barriers specifically relating to ergonomics of performing heel lance while babies are breastfed or held skin-to-skin.

	Not at all applicable (1)	Somewhat applicable (2)	Neutral (3)	Applicable (4)	Very applicable (7)
To what extent is this barrier applicable to your own practices? (1)	○	○	○	○	○

Q14 What other barriers or challenges exist that either prevent you or hinder you from facilitating breastfeeding or skin-to-skin care during heel lance?
Select all that apply.

- ☐ Baby is too critically ill (2)
- ☐ Too time consuming (3)
- ☐ Does not fit schedule (4)
- ☐ No suitable furniture / equipment (5)
- ☐ Too complicated (6)
- ☐ Does not seem to help the baby (7)
- ☐ Fears of baby choking (8)
- ☐ Parents are not present (9)
- ☐ Parents prefer not to be involved (10)
- ☐ Not enough room (11)
- ☐ Prefer not to be observed while doing heel lance (12)
- ☐ No policy or guidelines (13)
- ☐ Not routine practice (14)
- ☐ Other staff do not facilitate use of the strategies (15)
- ☐ Do not think it is necessary (16)
- ☐ Not confident with how to do it (17)
- ☐ Insufficient education regarding these practices (18)

☐ Other: (Please specify) (19) ____

Page Break

Q15 What factors support you or encourage you to facilitate breastfeeding or skin-to-skin care during newborn heel lance?   Select all that apply.

- ▢ Helps the baby (2)
- ▢ Complies with BFI (Baby-Friendly Initiative) (3)
- ▢ Baby medically stable (4)
- ▢ Baby bleeds well (5)
- ▢ Blood collection more efficient (6)
- ▢ Family centered practice (7)
- ▢ Parents ask to use these strategies (8)
- ▢ Parents wish to be involved (9)
- ▢ Sufficient room (10)
- ▢ Suitable furniture and equipment available to facilitate positioning (11)
- ▢ Confident in doing procedure (12)
- ▢ Practice supported by policy (13)
- ▢ Routine practice when possible (14)
- ▢ Other staff use the strategies (15)
- ▢ Consider it necessary to use the strategies (16)
- ▢ Parents present (17)
- ▢ Other: (Please specify) (18)

____

Page Break

Q16 How applicable is this video to your practice?

	Not at all applicable (1)	Not applicable (2)	Neutral (3)	Applicable (4)	Very applicable (5)
Applicability to your practice (1)	○	○	○	○	○

Q17 Comments

____

Q18 Was the amount of time it took to view this video acceptable?

	Very unacceptable (1)	Somewhat unacceptable (2)	Neutral (3)	Somewhat acceptable (4)	Very acceptable (5)
Acceptability of time to view (1)	○	○	○	○	○

Q19 Comments

____

Page Break

Q20 After viewing the Be Sweet to Babies ‘Ergonomics’ video, how likely do you think you will be to facilitate breastfeeding or skin-to-skin care during non-urgent heel lance procedures?

	Very unlikely (1)	Somewhat unlikely (2)	Neutral (3)	Somewhat likely (4)	Very likely (5)
Likelihood (1)	o	o	o	o	o

Q21 Comments

____

Q22 How likely would you be to recommend the Be Sweet to Babies ‘Ergonomics’ video to other staff?

	Very unlikely (1)	Somewhat unlikely (2)	Neutral (3)	Somewhat likely (4)	Very likely (5)
Likely to recommend video (1)	o	o	o	o	o

Q23 How effective do you think this video will be in increasing use of breastfeeding or skin-to-skin care during heel lance procedures?

	Not at all effective (1)	Not very effective (2)	Moderately effective (3)	Very effective (4)	Extremely effective (5)
Effectiveness (1)	o	o	o	o	o

Page Break

Q24 Further comments:
Feel free to add any comments on the ergonomics video, or any comments you have about using breastfeeding, skin-to-skin care, or sucrose for pain management.

____

Page Break

End of Block: video

Start of Block: Maternity Services-specific; if "Healthy newborns in Maternity Services" at Q2

Q26 How many years have you worked primarily in a maternity unit?

- Less than 1 (1)
- 1-5 (2)
- 6-10 (3)
- 11-15 (4)
- 16-20 (5)
- More than 20 (6)

Q27 What best describes your current employment status in your role in a maternity unit?

- Casual (1)
- Part time (2)
- Full time (3)

End of Block: Maternity Services-specific; if "Healthy newborns in Maternity Services" at Q2

Start of Block: neonatal nurse-specific; if "Sick newborns in Neonatal Services" at Q2

Q25 In which area do you predominantly work?

- ◯ Neonatal intensive care (NICU) (1)
- ◯ Special care nursery (SCN) (2)
- ◯ High dependency unit (HDU) (3)
- ◯ Other: Please specify below (4)

____

Q26 How many years have you worked in a neonatal unit caring for sick newborns?

- ◯ Less than 1 (1)
- ◯ 1-5 (2)
- ◯ 6-10 (3)
- ◯ 11-15 (4)
- ◯ 16-20 (5)
- ◯ More than 20 (6)

Q27 What best describes your current employment status in your role in a neonatal unit?

- ◯ Casual (1)
- ◯ Part time (2)
- ◯ Full time (3)

End of Block: neonatal nurse-specific; if "Sick newborns in Neonatal Services" at Q2

Start of Block: Demographics block - for all

Q28 What is your current registration? (select all that apply)

- ☐ Registered Nurse (3)
- ☐ Registered Midwife (7)
- ☐ Enrolled Nurse (8)
- ☐ Other (please specify) (10) ________________

Q29 a) Post-Graduate Certificate

- ☐ Neonatal intensive care (1)
- ☐ Paediatric (2)
- ☐ Paediatric intensive care (3)
- ☐ Other (please specify) (4) ________________

Q30 What is your highest academic qualification?

- ○ Bachelor of Nursing / Bachelor of Midwifery (1)
- ○ Post Grad Dip/Cert (3)
- ○ Masters by Coursework / Minor Thesis (4)
- ○ MPhil (research thesis) (5)
- ○ PhD / Clinical Doctorate (6)

Q31 Have you completed other relevant qualifications? (select all that apply).

Q32 b) Post-Graduate Diploma

- ▢ Neonatal intensive care (1)
- ▢ Paediatric (2)
- ▢ Paediatric intensive care (3)
- ▢ Other (please specify) (4) ________________

Q33 c) Lactation qualification

- ○ IBCLC certified (1)

Q34 What is your current **primary** role? (Select one only)

- Graduate nurse (in first 12 months of practice) (3)
- Graduate midwife (in first 12 months of practice) (4)
- Graduate nurse and midwife (in first 12 months of practice) (5)
- Clinical nurse specialist (9)
- Clinical midwife specialist (11)
- Caseload midwife (12)
- Lactation consultant (14)
- Research nurse and/or midwife (15)
- Clinic co-ordinator (16)
- Clinical support nurse (17)
- Clinical support midwife (18)
- Clinical educator (19)
- Care manager (20)
- Associate unit manager (22)
- Clinical nurse consultant/ Clinical midwife consultant (23)
- Team leader/ Unit manager (24)
- Other: (please specify) (25) ____

Q35 What state do you primarily work in?

- ○ Victoria (1)
- ○ New South Wales (2)
- ○ Queensland (3)
- ○ South Australia (4)
- ○ Western Australia (5)
- ○ Tasmania (6)
- ○ Northern Territory (7)
- ○ Australian Capital Territory (8)

End of Block: Demographics block - for all
